# The Self-Interaction of a Nodavirus Replicase Is Enhanced by Mitochondrial Membrane Lipids

**DOI:** 10.1371/journal.pone.0089628

**Published:** 2014-02-25

**Authors:** Yang Qiu, Zhaowei Wang, Yongxiang Liu, Yajuan Han, Meng Miao, Nan Qi, Jie Yang, Hongjie Xia, Xiaofeng Li, Cheng-Feng Qin, Yuanyang Hu, Xi Zhou

**Affiliations:** 1 State Key Laboratory of Virology, College of Life Sciences, Wuhan University, Wuhan, Hubei, China; 2 State Key Laboratory of Pathogen and Biosecurity, Beijing Institute of Microbiology and Epidemiology, Beijing, China; Chinese Academy of Sciences, Wuhan Institute of Virology, China

## Abstract

RNA replication of positive-strand (+)RNA viruses requires the protein-protein interactions among viral replicases and the association of viral replicases with intracellular membranes. Protein A from Wuhan nodavirus (WhNV), which closely associate with mitochondrial membranes, is the sole replicase required for viral RNA replication. Here, we studied the direct effects of mitochondrial membrane lipids (MMLs) on WhNV protein A activity *in vitro*. Our investigations revealed the self-interaction of WhNV protein A is accomplished via two different patterns (i.e., homotypic and heterotypic self-interactions via different interfaces). MMLs stimulated the protein A self-interaction, and this stimulation exhibited selectivity for specific phospholipids. Moreover, we found that specific phospholipids differently favor the two self-interaction patterns. Furthermore, manipulating specific phospholipid metabolism affected protein A self-interaction and the activity of protein A to replicate RNA in cells. Taken together, our findings reveal the direct effects of membrane lipids on a nodaviral RNA replicase.

## Introduction

One universal feature of positive-strand (+)RNA viruses is the assembly of their viral RNA replication complexes (vRCs), including viral replicase proteins, viral RNA, and host proteins, on host intracellular membranes [1]–[3]. During viral RNA replication, these viruses often induce specific intracellular membrane remodeling and lipid biosynthesis modifications via viral replicases [4]–[6]. On the other hand, lipids are major components of intracellular membranes, as they control membrane fluidity and plasticity [6], [7], and virus-induced modifications of lipid biosynthesis are closely linked to the formation and function of vRCs [2].

The viral protein-protein interaction is important for (+)RNA viruses replication [1]. Most (+)RNA viruses encode multiple viral proteins, which work together for the vRCs formation and function [8]–[13]. Although some replication proteins from many viruses have activity as individual units, they still require self-interaction/oligomerization for the complete functionality [14]–[17].

Nodaviruses (family *Nodaviridae*) are (+)RNA viruses that contain a bipartite genome consisting of two nonpolyadenylated RNAs, RNA1 (∼3.1 kb) and RNA2 (∼1.4 kb), which encode protein A, the RNA-dependent RNA polymerase (RdRP) [18] and capsid precursor protein α [19], respectively. A subgenomic RNA3 (sgRNA3), which is not encapsidated into virion, is synthesized during RNA1 replication and encodes nonstructural protein B2, a viral suppressor of RNA silencing [20].

In contrast to many (+)RNA viruses such as bromovirus, flavivirus, picornavirus and tombusvirus, in which a set of viral RNA replicase proteins synthesizes their RNA genomes, nodaviruses encode a sole RNA replicase protein, protein A, for viral RNA replication [1], [21]. This feature renders nodaviruses such as Flock House virus (FHV) and Wuhan nodavirus (WhNV) well-recognized and simplified models for studying viral RNA replication [22]–[27]. Previous studies of FHV, the most extensively studied member of the *Nodaviridae* family, revealed that FHV protein A contains multiple activities including synthesizes RNA, mitochondrial membrane association and self-interaction [22]. Disrupting the self-interaction of FHV protein A by the point mutations revealed that FHV protein A self-interaction is important for its function [28]. Moreover, the domains responsible for FHV protein A self-interaction include the *trans*-membrane regions, implying the correlation between membranes and protein A self-interaction [28], [29].

Multiple lines of evidence indicated that intracellular membranes, particularly membrane lipids, mediate FHV RNA protein A function. FHV protein A is a lipid-binding protein with particular affinity for specific anionic phospholipids, which may mediate the protein A-membrane interactions required for vRCs assembly [30]. The *in vitro* study showed that complete replication activity of FHV vRCs isolated in membrane fraction is disrupted by membrane-disrupting detergents, and can be augmented by the addition of exogenous phospholipids [31], [32]. Moreover, the genes involved in the synthesis of phosphatidylcholine play an important role in FHV RNA replication in *Drosophila* cells [33]. Inhibition of fatty acid synthesis using cerulenin resulted in the block of FHV RNA replication in *Drosophila* cells [34]. However, whether membrane lipids directly mediate nodaviral RNA protein A self-interaction is not well understood.

As a virus closely related to FHV, WhNV has been well characterized and provides novel insights for nodaviral subgenomic RNA replication [26] and RNA silencing suppression [35], [36]. Moreover, WhNV protein A can initiate RNA synthesis via *de novo* mechanism and contains a terminal nucleotidyl transferase activity [37]. Previous study showed that the activity of WhNV protein A to associate with mitochondrial membranes is closely linked with its activity for recruitment and stabilization of viral genomic RNA templates [38], suggesting the direct role of membrane lipids in WhNV protein A function. In this study, we focused on the effects of membrane lipids on WhNV protein A self-interaction. We expressed WhNV protein A *in vitro*, and isolated mitochondrial membrane lipids (MMLs) from mitochondrial outer membrane. Our study reveals that WhNV protein A is self-interacted and MMLs directly mediate protein A self-interaction in many aspects.

## Materials and Methods

### Plasmids

Standard procedures were used for restriction nuclease digestion and plasmid DNA construction and purification. To analyze WhNV protein A activity in cells, protein A ORF and RNA1 was inserted into pAC5.1/V5-His B vector (Invitrogen, Carlsbad, CA). Plasmids for the purification of MBP fusion protein A were constructed by inserting protein A ORF into pMAL-c2X (New England BioLabs, Ipswich, MA). For *in vitro* translation, WhNV and FHV protein A ORF was inserted into pET-28a (*Novagen, Germany*), respectively. Mutations were introduced into protein A ORF via PCR-mediated mutagenesis as described previously [26], [38]. The oligonucleotides used in this study are shown in Table 1.

**Table 1:** Oligonucleotides used in this work.

*Primers*	Sequences (5′ to 3′)
pA_ GAA_-His-R1	CTAAGCGTAATCTGGAACATCGTATGGGTAGCTTAAGGAACTATTCTTAAAGACT
pA_ GAA_ -His-R2	**GCGGCCGC**CTAAGCGTAATCTGGAACATCGTATGGGTAGC(NotI)
pA_ GAA_ -HA-R1	CTAATGATGATGATGATGATGGCTTAAGGAACTATTCTTAAAGACTAGAG
pA_ GAA_ -HA-R2	**GCGGCCGC**CTAATGATGATGATGATGATGGCTTAAGGAAC(NotI)
1–1014 MBP/protA-F	**GGATCC**ATGGTGTCAGTAATCAAGACAATAGTCG(BamH I)
1–1014 MBP/protA-R	**GTCGAC**TTAGCTTAAGGAACTATTCTTAAAGACTAGAGTTTCG(SalI)
1–254 MBP/protA-R	**GTCGAC**TTAGTTATTCTCAAAACGGTAAGCGAAC(SalI)
1–480 MBP/protA-R	**GTCGAC**TTATTTCCAGCAAACAAGGCTGGTTGTG(SalI)
1–659 MBP/protA-R	**GTCGAC**TTAGTGTAATCGCCTTCTTCTAATTCG(SalI)
1–839 MBP/protA-R	**GTCGAC**TTATCCATTTTTGAACTTCTTCTTGG(SalI)
255–1014 MBP/protA-F	**GGATCC** GAGATAGTGTATAACGTAACAGGTG (BamH I)
481–1014 MBP/protA-F	**GGATCC**AAAGTACGGAATGTAACAAAGTTTCC(BamH I)
660–1014 MBP/protA-F	**GGATCC**CTATATAACCAAATATACAAACAAC(BamH I)
840–1014 MBP/protA-F	**GGATCC**ACGGGAGAAGAACAATATCGCTGC(BamH I)
His/control-F	**GTCGAC**GCCACCATGGTGAGCAAGGGCGAGGAG(SalI)
His/control-R	**GCGGCCGC**TTACTTGTACAGCTCGTCCATGCC(NotI)
1–1014 His/protA-F	**GTCGAC**ATGGTGTCAGTAATCAAGACAATAGTCG(SalI)
1–1014 His/protA-R	**GCGGCCGC** TTAGCTTAAGGAACTATTCTTAAAGACTAGAGTTTCG (NotI)
1–254 His/protA-R	**GCGGCCGC**TTAGTTATTCTCAAAACGGTAAGCGAAC(NotI)
1–480 His/protA-R	**GCGGCCGC**TTATTTCCAGCAAACAAGGCTGGTTGTG(NotI)
1–659 His/protA-R	**GCGGCCGC**TTAGTGTAATCGCCTTCTTCTAATTCG(NotI)
1–839 His/protA-R	**GCGGCCGC**TTATCCATTTTTGAACTTCTTCTTGG(NotI)
255–1014 His/protA-F	**GTCGAC**GAGATAGTGTATAACGTAACAGGTG(SalI)
481–1014 His/protA-F	**GTCGAC**AAAGTACGGAATGTAACAAAGTTTCC(SalI)
660–1014 His/protA-F	**GTCGAC**CTATATAACCAAATATACAAACAAC(SalI)
840–1014 His/protA-F	**GTCGAC**ACGGGAGAAGAACAATATCGCTGC(SalI)
1–254/M1-F	CGCCCTACGTTTTACCGCAGCTGCAGCGGCGTGGAATTGCTGGACTAT
1–254/M1-R	ATAGTCCAGCAATTCCACGCCGCTGCAGCTGCGGTAAAACGTAGGGCG
1–254/M2-F	GAATATCTGGTCCGTGCTGTTGCACGTGCTGGTGCCACACCATATGTAGTCTC
1–254/M2-R	GAGACTACATATGGTGTGGCACCAGCACGTGCAACAGCACGGACCAGATATTC
FHV-protein A-F	**TCTAGA**ATGACTCTAAAAGTTATTCTTGGAGAACACCAG(XbaI)
**FHV-protein A-R**	**GTCGAC**TCACTTCCGGTTGTTGGAAGGCTGTGGCTGAGCTCC(SalI)

Sequence specific primers are designed according to Genbank no. AY962576 (WhNV RNA1). Characters in bold indicate restriction endonuclease sites, and the types are shown in brackets.

### Cells and Transfection

Pr-E cells, which is derived from *Pieris rapae* larvae, the natural host of WhNV, and was successfully utilized to study WhNV RNA replication previously (Qiu et al., 2011; Qiu et al., 2013), were maintained at 27°C in Grace’s medium (Gibco, Carlsbad, CA, USA) supplemented with 10% fetal bovine serum (Gibco). DNA plasmids were transfected into cells using FuGENE HD transfection reagent (Roche, Basel, Switzerland) according to the manufacturer’s protocol. All subsequent assays were performed 36 hrs after transfection except where indicated otherwise.

### WhNV *trans*-replication System

WhNV *trans*-replication system was previously established to study WhNV RNA replication [26], [38]. Briefly, we constructed two WhNV RNA1 mutants based on pAC1E plasmid, in which an EGFP open reading frame (ORF) is inserted at the 3′ end of RNA1 sequence [38]. The plasmid pAC1E is functional template for RNA1 replication (the transcribed and replicated products are labeled as “RNA1E”), but the ORF of protein A is closed by the mutation of the start codon [38]. WhNV protein A is provided by the plasmid pA, which the RdRp activity is remained but the ability for replication as RNA template is destroyed by deleting the 5′ and 3′ untranslated regions [38]. This WhNV *trans*-replication system, in which the RNA1 template and protein A mRNA are separately provided by two plasmids, was successfully used to study WhNV RNA replication and RNA recruitment/stabilization [38]. The assay was tested in Pr-E cells, 36 hrs after transfecting with the indicated plasmids, cells were collected and total RNA was separated, and 2 µg of total RNA was analyzed by Northern blotting.

### Western Blot Analysis and Antibodies

The proteins extracted from cells were subjected to 10% SDS-PAGE and Western blot analysis as previously described [26], [38]. Unless otherwise indicated, the anti-MBP polyclonal antibody was purchased from New England BioLabs, and the other primary and secondary antibodies were purchased from Proteintech, Chicago, IL, USA.

### RNA Extraction and Northern Blot Analysis

Total RNA was extracted from cells using TRIzol reagent (Invitrogen) and digested with RQ1 RNase-free DNase I (Promega, Madison, WI, USA) as previously described [26], [38]. For Northern blot analysis, 2 µg of each RNA sample was analyzed via Northern blot analysis as previously described [26], [38]. The probes for (+) and (−) EGFP were complementary to the entire EGFP sequences. All probes were labeled with DIG-UTP (Roche) for *in vitro* transcription; the corresponding oligonucleotides are shown in Table 1.

### Purification of Protein A and its Derivatives

The expression and purification of recombinant WhNV protein A and its derivatives were carried out as previously described [35]–[37], [39]. Briefly, to obtain soluble recombinant protein, Maltose-binding protein (MBP)-tagged full-length protein A and its mutants as well as the negative control protein MBP were expressed in *Escherichia coli* strain TB1 at 20°C in the presence of 0.2 mM IPTG. Cell pellets were resuspended in binding buffer (20 mM Tris-HCl [pH 7.4], 200 mM NaCl, 1 mM EDTA, 10 mM 2-Mercaptoethanol) supplemented with 1.5% Triton-X 100 and protease inhibitors cocktail (Sigma, St. Louis, Mo, USA). Cells were lysed by sonication and then debris was removed by centrifugation for 30 min at 11,000 ×g. The proteins in the supernatant were purified using amylose resin (New England BioLabs) according to the manufacturer’s protocol and concentrated using Amicon Ultra-15 filters (Millipore, Schwalbach, Germany), and the buffer was exchanged to the hypotonic buffer (1 mM HEPES [pH 7.4], 0.1 mM EDTA, 15 mM NaCl, 1 mM DTT). For *in vitro* translation, His-tagged proteins were translated using nuclease-treated rabbit reticulocyte lysates (Promega) according to the manufacturer’s protocol. All proteins were quantified via a UV-visible spectrophotometer (Shimadzu, Kyoto, Japan).

### Mitochondrial Membrane Lipids and Liposomes

Mitochondrial outer membranes were isolated from Pr-E cells by mechanical disruption and differential centrifugation as previously described [40], [41], and then determined by immuno-detections (Fig. S1). Subsequently, the purified outer mitochondrial membranes were treated with 0.1 mg/ml proteinase K (Sigma) for 10 min in hypotonic buffer supplemented with 1.5% Triton-X 100 to dissolve integral membrane proteins. MMLs were then reisolated by centrifugation at 12,000 × g for 20 min and resuspended in hypotonic buffer. MMLs were further purified and concentrated by using Amicon Ultra-15 filters (Millipor). Lipids were obtained from Sigma in the highest purity grades available: 1,1′,2,2′-tetraoleoyl cardiolipin (CL), 1,2-dioleoylsn-glycero-3-phosphate (PA), 1,2-dioleoyl-*sn*-glycero-3-[phospho-*rac*-(1-glycerol)] (PG), 1,2-dioleoyl*sn*-glycero-3-[phospho-L-serine] (PS), 1,2-dioleoyl-*sn*-glycero-3-phosphocholine (PC) and 1,2-dioleoyl-*sn*-glycero-3- phosphoethanolamine (PE). The liposomes were prepared as described [30]–[32], [42]. Briefly, the purchased lipids were dissolved and mixed in chloroform/methanol (2∶1) at 10 mg lipid per 1 ml organic solvent. The mixture was dried under nitrogen and lyophilized to remove any traces of solvent. The dry film was hydrated with 20 mM HEPES buffer at pH 7.4 by vortexing overnight at 4°C. The purified MMLs and liposomes were quantified by Bradford protein assay (Bio-Rad) using a UV-visible spectrophotometer (Shimadzu).

### Protein flotation Assays

For MML-binding assays, MBP-tagged protein A or its derivatives (10 pmol each) was incubated with MMLs (50 µg per 100 µl reaction mixture) in flotation buffer (50 mM HEPES [pH 7.4], 50 mM KCl, 2 mM MgCl_2_, 1 mM DTT) for 1 h at room temperature. After the incubation, the reaction mixtures were diluted with 4 volumes of flotation buffer, and Nycondenz (Sigma) was added to the mixtures to a final concentration of 37.5% (wt/vol), and samples were loaded under a 5% to 25% discontinuous Nycondenz gradient and centrifuged to equilibrium at 100,000 × g for 20 hrs at 4°C in a Beckman Coulter SW40 rotor. After centrifugation, the gradient was divided into two fractions including the upper half of the gradient (low-density fraction, LD) and the lower half of the gradient (high-density fraction, HD). Protein samples were isolated from each fraction via centrifugation at 180,000× g in a Beckman Coulter SW40 rotor for 3 hrs and then analyzed via Western blotting.

### Pull-down Assays

MBP pull-down assays were performed with recombinant MBP fusion proteins and His-tagged proteins as previously described [35], [36]. Briefly, amylose resin (New England Biolabs) was added to the reaction mixtures containing MBP fusion and His fusion proteins and then incubated at room temperature for 4 hrs. To test the stimulating effects of MMLs on protein A self-interaction, MMLs were added (0.1 to 10 µg MMLs per 1 µl reaction mixture) to the reaction mixtures. After subsequent washing and collection, proteins that bound to the amylose resin were subjected to Western blot analysis. Immunoblotting signals were quantified and plotting results against standard curves from immunoblotting of serially diluted samples (data not shown).

### Chemical Cross-linking Assays

Chemical cross-linking assays were performed as previously described [36]. MBP-tagged proteins were cross-linked in cross-linking buffer (10 mM HEPES [pH 7.4], 50 mM MgCl2, 1 mM DTT, 1% glycerol, and 0.03% [vol/vol] glutaraldehyde) for 30 min. The complexes were then analyzed via 10% SDS-PAGE.

### PA Inhibitor and Cells Viability Assays

5-fluoro-2-indolylde-chlorohalopemide (FIPI) (Sigma) was used to inhibit PA production as previously described [43]. Briefly, 12 hrs after transfection, cells were treated with 75 nM FIPI in DMSO and incubated for another 24 hrs. Then cells were collected, and divided into two equal fractions. One fraction was used for Co-IP experiments as described below, and the other fraction was used for total RNA extraction and following analyzing by Northern blot as described above. Cell viability assays were performed using MTT (Sigma) as previously described [33].

### Coimmunoprecipitation Assays

Coimmunoprecipitation (co-IP) assays were performed as previously described [35], [36]. Briefly, 36 hrs after transfection and FIPI treatment, cells were lysed with NETN buffer [20 mM Tris-HCl (pH 7.4), 150 mM NaCl, 1 mM EDTA, and 0.5% NP-40] for 20 min at 4°C in the presence of protease inhibitors cocktail (Sigma). Lysates were clarified at 12,000 rpm for 10 min at 4°C, and then postnuclear lysates were precleared via incubation with protein-G agarose beads (Roche) coupled to goat anti-mouse IgG and then incubated with antibodies (mouse anti-His antibody, mouse anti-HA antibody, or control mouse anti-FLAG antibody) at room temperature for 4 hrs. The antibody-bound complexes were captured, washed, and then subjected to SDS-PAGE and Western blotting analysis with rabbit anti-His antibody or rabbit anti-HA antibody.

### PA Determination

Total PA content was determined using a modified phospholipase D-based enzymatic method [44]. Briefly, cells were detached by treatment with PBS plus 5 mM EDTA and resuspended in 1× PBS buffer containing 1 mM MgCl_2_, 1 mM CaCl_2_, 5 mM glucose, and 0.2% BSA. Cells were then immediately frozen and the total cellular lipids were incubated first with lipoprotein lipase of *Pseudomonas* sp (Sigma) then with glycerol-3-phosphate oxidase (Sigma) in the presence of horseradish peroxidase (Sigma) and Amplex Red (Invitrogen). Then the PA content was measured by fluorescence emission at 580 nm after excitation at 530 nm. A standard curve was generated using purchased and purified PA as described above PA detection was normalized to total protein content in the cellular sample determined before lipid extraction.

## Results

### Characterization of the Self-interaction of WhNV Protein A

**Figure 1:**
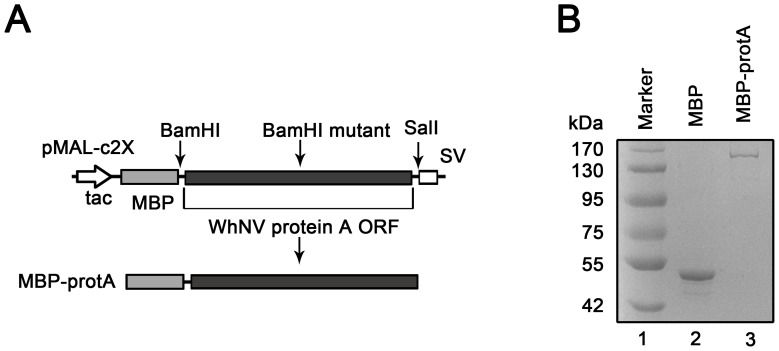
Expression of recombinant WhNV protein A. (A) Schematic representation of the expression strategy of WhNV protein A. Protein A ORF was cloned into pMAL-c2X and expressed as C-terminal fusion proteins with MBP (MBP-protA). To manipulate the vector, we mutated a BamH I restriction endonuclease site on protein A ORF sequences. (B) SDS-PAGE analysis of purified recombinant protein A from *E. coli*. Lane 1, Marker; lane 2, MBP protein alone; lane 3, MBP-protA.

To characterize the relationship between self-interaction of WhNV protein A and membrane lipids directly, MBP-tagged full-length (FL) protein A (MBP-protA) was expressed in *E. coli* and purified (Fig. 1A). MBP-protA was expressed at its expected molecular weight (around 158 kDa; Fig. 1B, lane 3), and MBP protein alone was expressed as the negative control under the same condition (Fig. 1B, lane 2).

**Figure 2:**
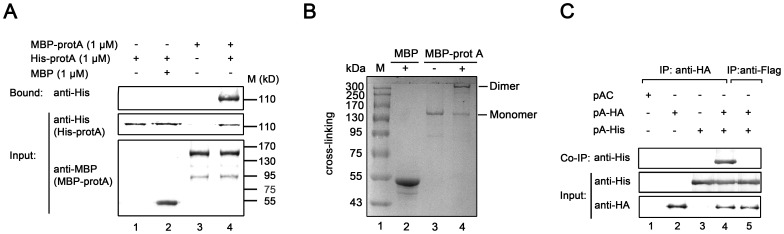
Characterization of WhNV protein A self-interaction. (A) Protein A exhibited self-interaction. Pull-down buffer alone (lane 1), MBP alone (lane 2) or MBP-protA (lanes 3 and 4, 1 µM each) was used to pull-down the *in vitro* translation His-tagged protein A (His-protA) (lanes 1, 2 and 4, 1 µM each) or the translation buffer (lane 3), and then subjected to Western blotting with anti-His antibody (Top). The input proteins were subjected to Western blotting with anti-His and anti-MBP antibodies, respectively, as shown in middle and bottom. The sizes of the molecular weight markers are indicated on the left in thousandths. (B) Cross-linking of MBP-protA. MBP alone (lane 2) or MBP-protA (lane 4) was incubated in a cross-linking buffer and then analyzed via 10% SDS-PAGE. The dimer and monomer form of MBP-protA are indicated. M, marker. (C) Self-interaction of protein A in cells. Pr-E cells expressing empty vector (lane 1) or either HA-tagged protein A (lane 2), His-tagged protein A (lane 3), or both (lanes 4 and 5) were harvested. Lysates were immunoprecipitated with either anti-HA antibody (lanes 1–4) or anti-FLAG antibody (lane 5) and probed via Western blotting with anti-His antibody. The middle and bottom panels present input of proteins with two tags, respectively.

To determine whether WhNV protein A can self-interact *in vitro*, we used 1 µM of MBP-protA to pull down 1 µM of His-tagged protein A (His-protA) that was expressed in nuclease-treated rabbit reticulocyte lysates (RRLs) *in vitro*. The bound complexes were analyzed by Western blots using anti-His antibody, and the input lysates were also detected using anti-His and anti-MBP antibodies, respectively. His-protA was efficiently pulled down by MBP-protA (Fig. 2A, lane 4), but it did not interact with negative control MBP protein alone (Fig. 2A, lane 2). The dimerization of protein A was further confirmed using MBP-protA cross-linking assay *in vitro* (Fig. 2B). MBP-protA was incubated in the chemical cross-linking for 20 min, and then the samples were analyzed via SDS-PAGE, revealing one band with molecular weight about 330 kDa (Fig. 2B, lane 4), indicating that protein A can form homodimer.

The protein A self-interaction was further confirmed via co-IP in Pr-E cells. As shown in Fig. 2C, Pr-E cells were transfected with either empty vector (pAC) (lane 1), a plasmid expressing protein A with C-terminal HA tag (pA-HA) (lane 2), a plasmid expressing protein A with a C-terminal His tag (pA-His) (lane 3), or with both pA-HA and pA-His (lane 4). After 36 h of transfection, cells were harvested, and protein complexes were immunoprecipitated with anti-HA antibody and following by Western blots with anti-His antibody. Protein A self-interaction was present in cells (Fig. 2C, lane 4), whereas no protein was immunoprecipitated with a control antibody anti-Flag (Fig. 2C, lane 5). Taken together, these results show that protein A can be self-interacted (homodimerized) *in vitro* and in cells.

### Characterization of the Fragments Responsible for WhNV Protein A Self-interaction and the Homotypic and Heterotypic Interactions among these Fragments

**Figure 3:**
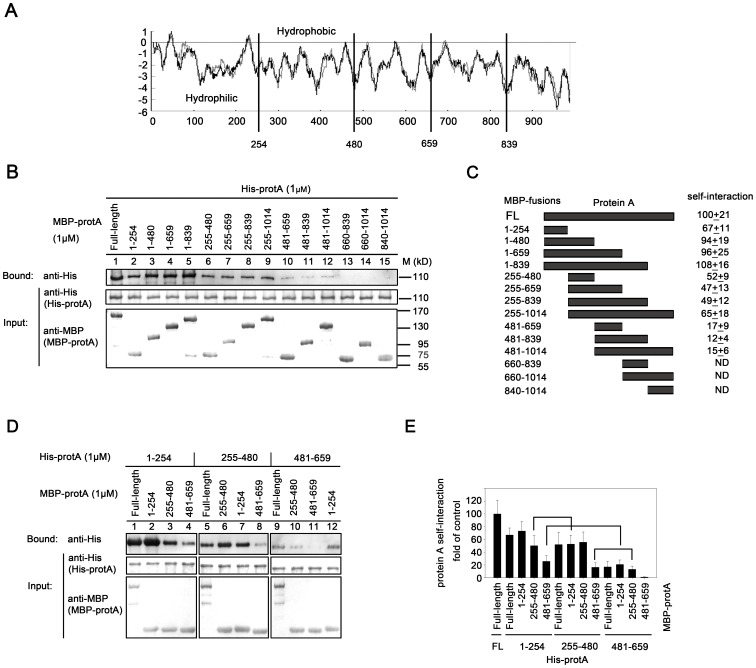
Characterization of the fragments responsible for WhNV protein A self-interaction and the homotypic and heterotypic interactions among these fragments. (A) Potential hydrophobic regions of WhNV protein A. (B) MBP-tagged protein A fragments (1 µM each) were used to pull-down FL His-protA (1 µM). The sizes of the molecular weight markers are indicated on the left in thousandths. (C) Summary of MBP fusion proteins and their activities to interact with His-protA, representing the results shown in (B). The self-interaction efficiency of protein A fragments was measured as the percentage of protein A FL self-interaction. ND, not detected. FL, full-length. (D) The self-interacting fragments form homotypic and heterotypic self-interactions. MBP-tagged protein A fragments (1 µM each) were used to pull-down His-tagged protein A fragments (1 µM each). (E) Summary of the homotypic and heterotypic interactions of protein A, representing the results shown in (D).

We sought to determine the fragments required for protein A self-interaction. Thus, a series of MBP-protA fragments were produced according to the hydrophobicity of protein A amino acid (aa) sequences (Fig. 3A) and then used to pull down His-protA (Fig. 3B). The self-interaction efficiency of these protein A fragments was measured as the percentages of the self-interaction of FL to FL protein A (i.e., MBP-protA pulls down His-protA; Fig. 3B, lane 1; Fig. 3C, right, “FL”). We found that multiple fragments were required for protein A self-interaction. MBP-protA fragments aa 1–254, aa 255–480, and aa 481–659 exhibited 67%, 52%, and 17% self-interaction efficiencies to FL His-protA, respectively (Fig. 3B, lanes 2, 6, and 10; Fig. 3C, right). On the other hand, fragments aa 660–839, aa 660–1014, and aa 840–1014 did not contribute to protein A self-interaction (Fig. 3B, lanes 13–15; Fig. 3C, right). The elongation of aa 1–254 to aa 480, aa 659, or aa 839 resulted in an increase in self-interaction to a level comparable to that of FL to FL protein A self-interaction (94%, 96%, and 108%, respectively; Fig. 3B, lanes 3–5; Fig. 3C right). On the other hand, the elongation of aa 255–480 to aa 659, aa 839, or aa 1014 did not further affect protein A self-interaction (Fig. 3B, lanes 7–9; Fig. 3C, right). Similar results were observed when aa 481–659 was elongated to aa 839 or aa 1014 (Fig. 3B, lanes 11 and 12; Fig. 3C, right). Taken together, we conclude that aa 1–254 and aa 255–480 are sufficient to mediate protein A self-interaction.

Given that protein A self-interaction is mediated at least by two distinct fragments, we also sought to determine whether the self-interaction is formed by homotypic (i.e., aa 1–254/1–254 and aa 255–480/255–480) and/or heterotypic (i.e., aa 1–254/255–480) interactions of these two fragments of protein A. To that end, we assessed the potential homotypic and heterotypic interactions using MBP-protA fragments aa 1–254, aa 255–480, and aa 481–659 to pull down *in vitro* translated His-protA fragments aa 1–254, aa 255–480, and aa 481–659, respectively. Various homotypic and heterotypic interactions were detected (Fig. 3D), and the results were graphed as the percentages of the FL to FL protein A self-interaction (Fig. 3E). The homotypic interactions of aa 1–254 and aa 255–480 were 73% and 55%, respectively, of the level of FL protein A self-interaction, whereas the homotypic interaction of aa 481–659 was very weak (2%). Heterotypic interactions between aa 1–254 and aa 255–480 were also detected (50–52%). Interestingly, although the homotypic self-interaction of aa 481–659 was minimal, the heterotypic interactions of this fragment with aa 1–254 and aa 255–480 were relatively substantial (30% and 22%, respectively), thereby implying that the aa 481–659 fragment mediates protein A self-interaction via facilitating heterotypic interactions (Fig. 3B). Taken together, these results show that both homotypic and heterotypic interactions of protein A fragments exist and act together to mediate protein A self-interaction.

### Mitochondrial Membrane Lipids Stimulate WhNV Protein A Self-interaction

**Figure 4:**
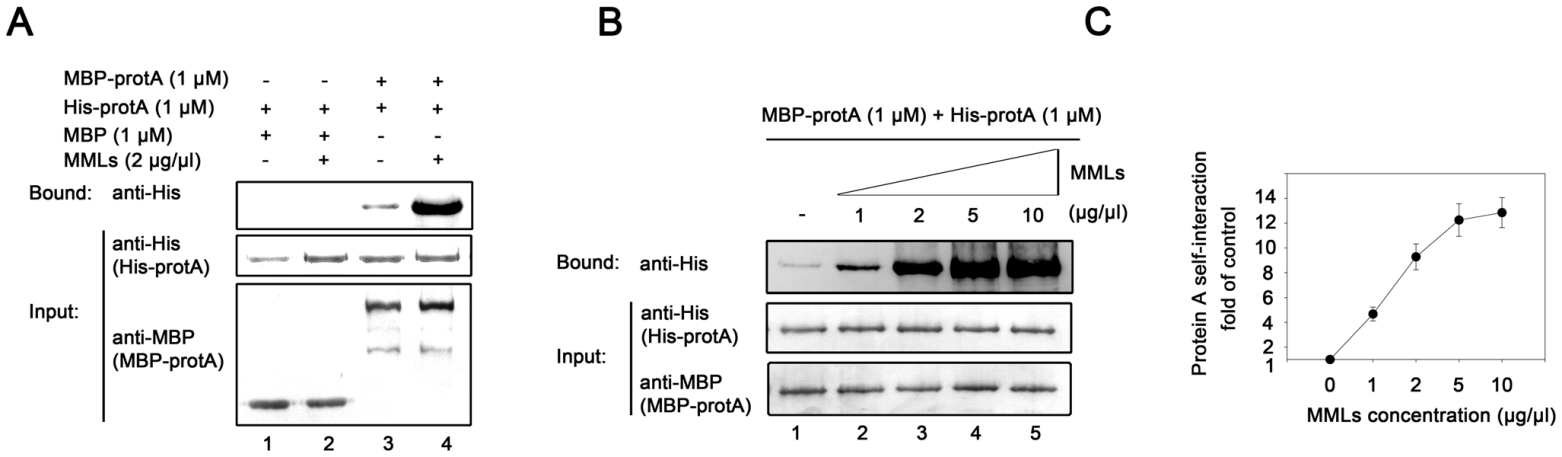
MMLs stimulate WhNV protein A self-interaction. (A) Protein A self-interaction is increased by MMLs. MBP-protA (lanes 3 and 4, 1 µM) or MBP alone (lanes 1 and 2) was used to pull down the His-protA (1µM) in the absence (lanes 1 and 3) or in the presence (lanes 2 and 4) of 2 µg/µl MMLs, and then subjected the pull-down products to Western blotting with anti-His antibody. (B–C) MMLs stimulate protein A self-interaction in a dose-response manner. Increasing concentrations (wt/vol) of MMLs were incubated with MBP-protA and His-protA (1µM each). The concentrations of MMLs are indicated above each lane. The self-interaction of protein A in the absence of MMLs is used as the control (1-fold). The increases in the self-interaction of protein A at each point concentration of MMLs are graphed as the fold of the control as shown in (C). Error bars represent S.D. values from at least three independently repeated experiments and the represent results were shown in (B).

Subsequently, we examined the direct effect of MMLs on the self-interaction of protein A under the same conditions described in Fig. 2A with the addition of 2 µg/µl MMLs. In the presence of MMLs, the ability of protein A to self-interact was substantially increased (Fig. 4A, lane 4), whereas MMLs had no effect on MBP alone (Fig. 4A, lane 2). To further confirm the stimulating effect of MMLs on protein A dimerization, we conducted a dose-response assay (Fig. 4B). As the concentration of MMLs increased, the self-interaction of protein A was gradually enhanced (Fig. 4B, “Bound”). Protein A self-interaction was stimulated about 4.6-fold at an MML concentration of 1 µg/µl, about 9-fold at an MML concentration of 2 µg/µl, about 12-fold at an MML concentration of 5 µg/µl, and then plateaued at an MML concentration of 10 µg/µl (Fig. 4C). Together, these results confirmed that MMLs promoted protein A self-interaction.

### Mitochondrial Membrane Lipids Stimulate WhNV Protein A Self-interaction by Promoting the Homotypic and Heterotypic Interactions of Protein A

**Figure 5:**
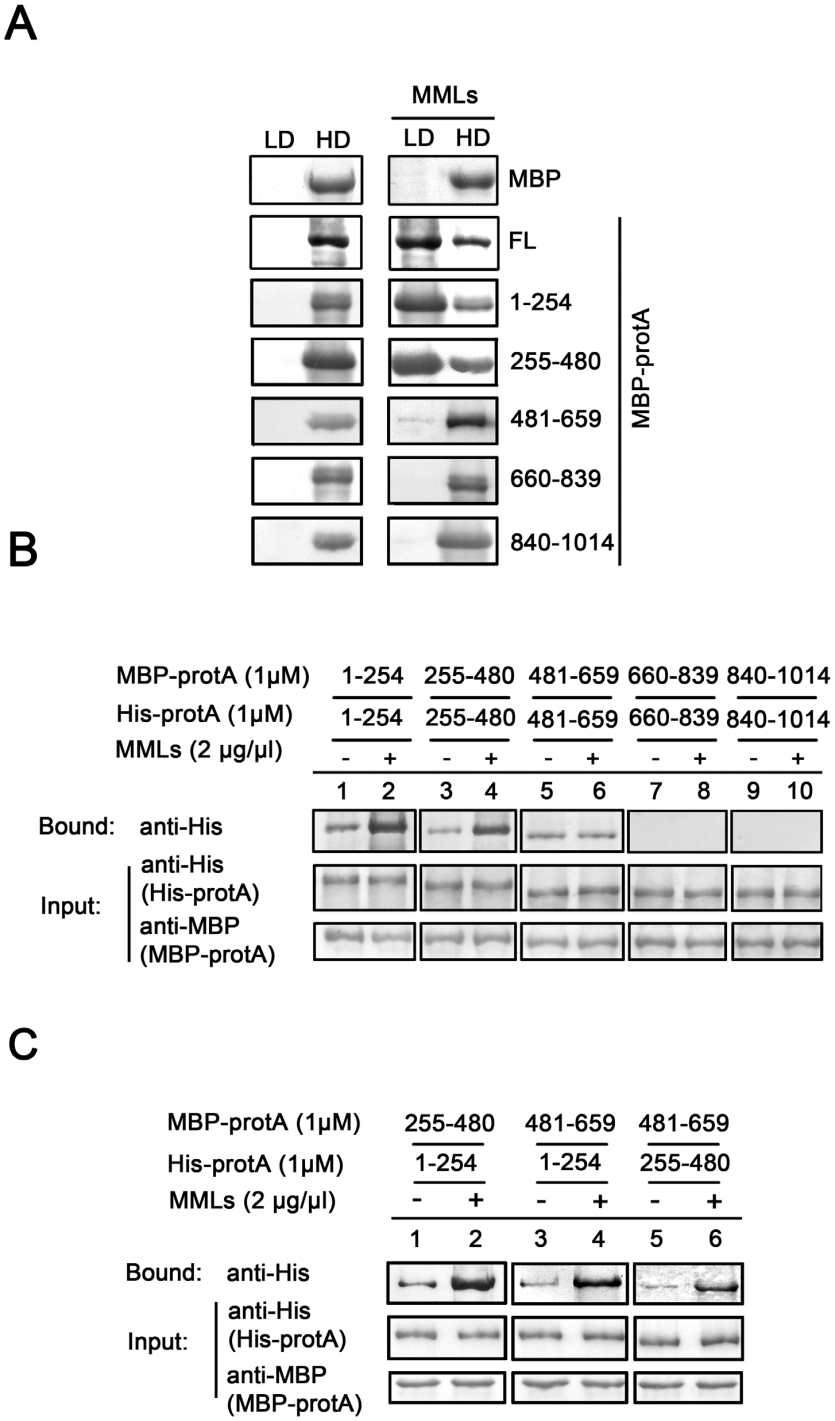
MMLs stimulate WhNV protein A self-interaction by promoting the homotypic and heterotypic interactions of protein A. (A) MBP-tagged protein A fragments were incubated without (left) or with the MMLs (right) and subjected to Nycodenz flotation. The LD and HD fractions were analyzed via Western blotting with anti-MBP antibody. (B–C) The effects of MMLs on different homotypic (B) and heterotypic (C) interactions of protein A.

After identifying the stimulating effects of MMLs on protein A self-interaction and the fragments responsible for protein A self-interaction, we next attempted to determine whether these fragments are responsible for protein A’s binding to MMLs. To this end, we incubated various protein A fragments in the presence or absence of MMLs and then subjected them to Nycodenz gradient flotation assays to examine their MML association (Fig. 5A). MBP alone was used as the negative control, thereby ruling out the possibility that MBP induces protein-MML interaction. The flotation gradients were divided into two fractions, LD and HD. As previously shown [38], the LD fractions represent the membrane-rich layers in the gradient, whereas the HD (non-membrane) fractions contain cytosolic soluble proteins. As shown in Fig. 5A, none of these fragments can be detected in LD fractions in the absence of MMLs, whereas a larger part of the aa 1–254 and aa 255–480 was recovered in LD fractions in the presence of MMLs. These results indicate that the two protein A fragments, aa 1–254 and aa 255–480, are responsible for the binding of protein A to MMLs. Given that these two fragments are also sufficient for protein A self-interaction, these results suggest that MMLs may interact with these fragments, stimulate the homotypic and heterotypic interactions of these fragments and subsequently promote the whole protein A self-interaction.

We sought to assess the effects of MMLs on the homotypic interactions of aa 1–254, aa 255–480, and aa 481–659. Of note, because of the weak homotypic interaction of aa 481–659, 10 more times samples were loaded than in Fig. 3B (lane 11) for a better observation. MMLs stimulated the homotypic interaction of aa 1–254 and aa 255–480 but not that of aa 481–659 (Fig. 5B, lanes 1–6). In addition, neither aa 660–839 nor aa 840–1014 homotypically interacted in the absence or presence of MMLs (Fig. 5B, lanes 7–10).

Subsequently, we determined the stimulating effects of MMLs on the heterotypic interactions among the aa 1–254, aa 255–480, and aa 481–659 fragments. All three types of heterotypic interactions were enhanced by MMLs (Fig. 5C). Interestingly, although the weak homotypic interaction of aa 481–659 was not stimulated by MMLs (Fig. 5C, lanes 5–6), the heterotypic interaction of aa 481–659 with aa 1–254 or aa 255–480 was efficiently stimulated by MMLs (Fig. 5C, lanes 3–6). In summary, the results of this set of experiments demonstrate that MMLs stimulate protein A self-interaction by enhancing both the homotypic and heterotypic interactions of the specific fragments of protein A.

### Characterization of the Stimulating Effect of Mitochondrial Membrane Lipids on Protein A Self-interaction Activity

**Figure 6:**
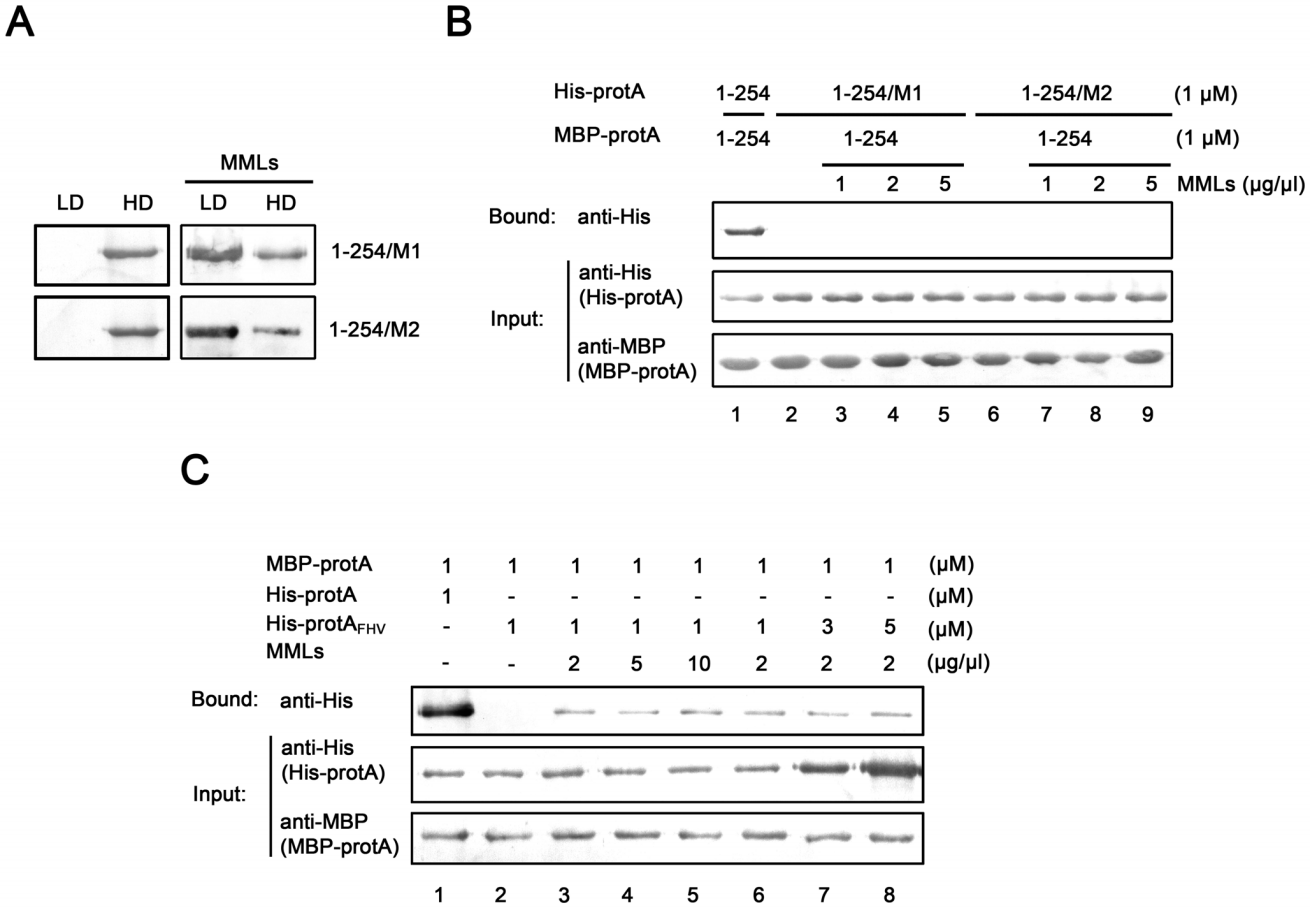
Characterization of the stimulating effect of MMLs on protein A self-interaction activity. (A) The *in vitro* translation His-protA fragments aa 1–254/M1 (K91A, W92A, and R93A) and aa 1–254/M2 (S163A, R165A and Y169A) were incubated without (left) or with the MMLs (right) and subjected to Nycodenz flotation. (B) MBP-protA fragment aa 1–254 was used to pull-down His-protA fragments aa 1–254/M1 (lanes 2–5) and aa 1–254/M2 (lanes 3–8) in the increasing concentrations of MMLs (lanes 3–5 and 7–8). The concentrations of MMLs are indicated above each lane. The wt protein A fragment aa 1–254 was used as the control (lane 1). (C) MBP-protA was used to pull-down 1 µM His-protA_FHV_ at the increasing concentrations of MML (lanes 3–5), or increasing concentrations of His-protA_FHV_ at the 2 µg/µl MMLs (lanes 6–8). The concentrations of His-protA_FHV_ and MMLs are indicated above. The WhNV protein A self-interaction was used as the control (lane 1).

Because WhNV protein A is a membrane binding protein, it is possible that protein A interacts with another protein A via a common lipid “bridge”. To test this possibility, we sought to define the sites critical for protein A self-interaction, mutate these regions without affecting the MML binding of protein A, and then determine the self-interaction of these mutants in the absence or presence of MMLs. To this end, amino acid substitutions were introduced into the aa 1–254 and were expressed in nuclease-treated RRLs; and in each mutant, the original amino acid was changed to alanine. We constructed multiple single-site mutations spanning aa 1–254; however, the triple-sites mutations completely lost their self-interacting activities. Then two aa 1–254 mutants, aa 1–254/M1 (K91A, W92A, and R93A) and aa 1–254/M2 (S163A, R165A and Y169A) were used to test their abilities to binding to MMLs and self-interact in the presence of MMLs, respectively. A shown in Fig. 6, aa 1–254/M1 and aa 1–254/M2 still contain the MML-binding property as being determined by Nycondenz gradient centrifugation (Fig. 6A), but completely lost their self-interacting activities (Fig. 6B, lanes 2 and 6). Furthermore, our result showed that the self-interactions of these mutants were unable to be stimulated by MMLs at various concentrations (Fig. 6B, lanes 3–5 and 7–9).

Furthermore, we used another MML binding protein, FHV protein A, to test if FHV protein A could also interact with WhNV protein A via the possible “bridging” effect of MMLs. MBP-protA was used to pull-down His-tagged FHV protein A (His-protA_FHV_) that was expressed in nuclease-treated RRLs *in vitro*. The interactions between WhNV protein As with MBP and His tags were used as the positive control (Fig. 6C, lane 1). As shown in Fig. 6C, MBP-protA can not interact with His-protA_FHV_ in the absence of MMLs (lane 2), showing that WhNV protein A and FHV protein A have no direct protein-protein interaction. The presence of 2 µg/µl MMLs resulted in a very weak interaction of these two proteins (compared lane 3 to lane 1, the positive control of WhNV protein A self-interaction at the same protein concentrations); moreover, either increasing the MML concentrations (Fig. 6C, lane 3–5) or increasing the His-protA_FHV_ concentrations (Fig. 6C, lanes 6–8) showed no stimulating effect on the weak indirect protein A_WhNV_-protein A_FHV_ interaction. These results indicate that binding to common lipid may contribute to but could not be the major cause for the stimulation on protein A self-interaction, since the indirect interaction through binding to MMLs is much weaker than the protein-protein interaction and can not be further enhanced by increasing the concentrations of MMLs or protein.

### Specific Anionic Phospholipids Stimulate Protein A Self-interaction

**Figure 7:**
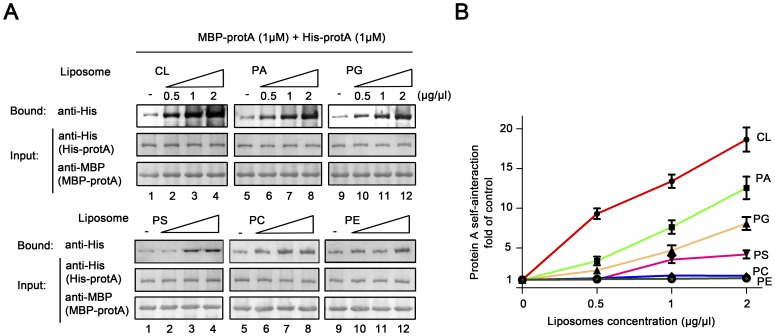
Specific anionic phospholipids stimulate protein A self-interaction. (A–B) MBP pull-down was performed on the increasing concentrations (wt/vol) of liposomes generated from specific purified phospholipids with MBP-protA-His-protA complex. The concentrations of liposomes are indicated above each lane. The self-interaction of protein A in the absence of liposomes is used as the control (1-fold). The increases in the self-interaction of protein A at each point concentration of liposomes are graphed as the fold of the control as shown in (E). Error bars represent S.D. values from at least three independently repeated experiments.

MMLs are composed of various specific phospholipids [7]. The various phospholipid compositions of intracellular membranes are the key determinants of the activities of membranes as well as membrane-associated proteins [7]. Thus, we further analyzed the self-interaction activity of protein A with liposomes that made of individual major outer mitochondria membrane phospholipids. A series of dose-response assays were performed to determine the effect of distinct liposomes on the self-interaction of protein A (Fig. 7A). And the data was graphed as the fold of the self-interaction of protein A without lipids. As shown in Fig. 7B, protein A self-interaction was substantially stimulated in the presence of increasing concentrations of CL and PA. PG and PS stimulated protein A self-interaction moderately, whereas PC and PE did not affect protein A self-interaction. These results indicate that protein A self-interaction is selectively stimulated by specific anionic phospholipids.

### Specific Anionic Phospholipids Favor Different Types of Self-interactions of Protein A aa 1–254 and aa 255–480

**Figure 8:**
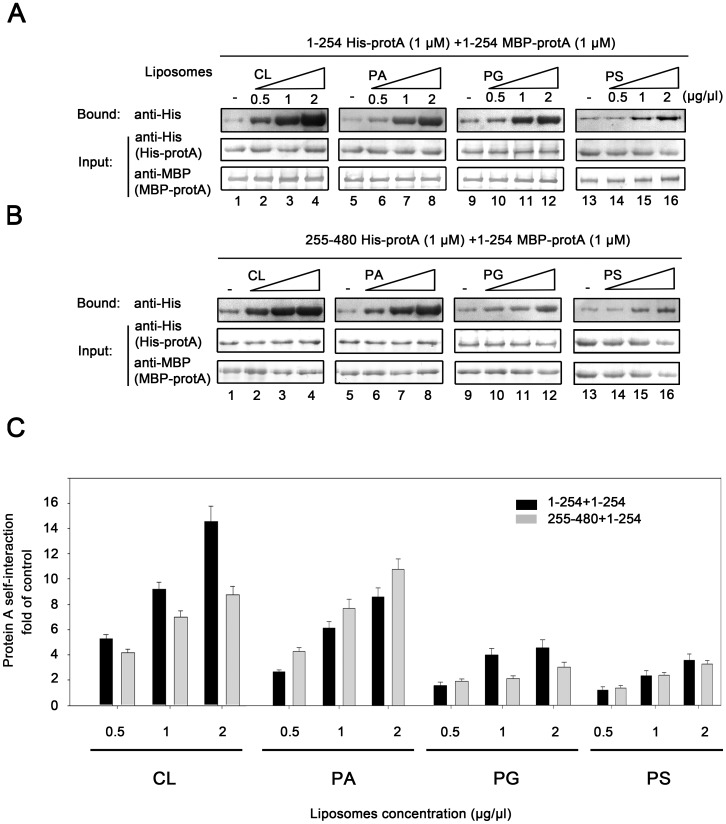
Specific anionic phospholipids favor different types of self-interactions of protein A aa 1–254 and aa 255–480. (A–C) The homotypic interactions of aa 1–254 and the heterotypic interactions of aa 1–254 and aa 255–480 were examined in the presence of the increasing concentrations of liposomes generated from CL, PA, PG, or PS, respectively. The homotypic or heterotypic interactions in the presence of different liposomes at each concentration are graphed as shown in (C). The homotypic (gray bar) and heterotypic (black bar) interactions in the absence of liposomes are used as the control (1-fold). The increase in homotypic or heterotypic interactions in the presence of different liposomes at each concentration is graphed as the fold of control. Error bars represent S.D. values from at least three independent experiments and the represent results were shown in (A–B).

Having shown that homotypic and heterotypic interactions exist during protein A self-interaction and that specific anionic phospholipids stimulate protein A self-interaction at various levels, we hypothesized that the homotypic and heterotypic interactions of protein A could be differentially mediated by specific anionic phospholipids. To test this hypothesis, we assessed the effects of various anionic phospholipids on the homotypic interactions of aa 1–254 and the heterotypic interactions of aa 1–254 and aa 255–480. MBP pull-down assays were conducted in the presence of liposomes containing increasing concentrations of CL, PA, PG, or PS (Fig. 8A and B). Increases in the levels of homotypic or heterotypic interactions at different concentrations of various liposomes were measured and graphed as the fold of interactions without lipids (Fig. 8C). Interestingly, our results revealed the different levels of homotypic and heterotypic interactions in the presence of different liposomes. As shown in Fig. 8C, as the liposome concentration increased, CL gradually favored the homotypic interactions (black bar is gradually higher than gray bar at each point concentrations of CL). According to PG, the homotypic and heterotypic interactions show no significant difference at the concentration of 0.5 µg/µl, but the homotypic interactions is stronger than the heterotypic interactions with the concentration of PG increased (1 and 2 µg/µl, black bar is higher than gray bar). While PA had an opposite effect and favored the heterotypic interactions all the time (gray bar is higher than black bar at all point concentrations of PA). Moreover, the homotypic and heterotypic interactions did not differ in the presence of increasing concentrations of PS. These results indicate that specific phospholipids favor different patterns of protein A self-interactions.

### Manipulation of Phospholipid Metabolism Affects Protein A-induced RNA Replication and Self-interaction in Cells

To further investigate the effects of MMLs, particularly changes in MMLs, on the functions of protein A in cells, we aimed to manipulate phospholipid synthesis in Pr-E cells to assess protein A activity with regard to membrane association, self-interaction, and RNA1/sgRNA3 replication in cells.

**Figure 9:**
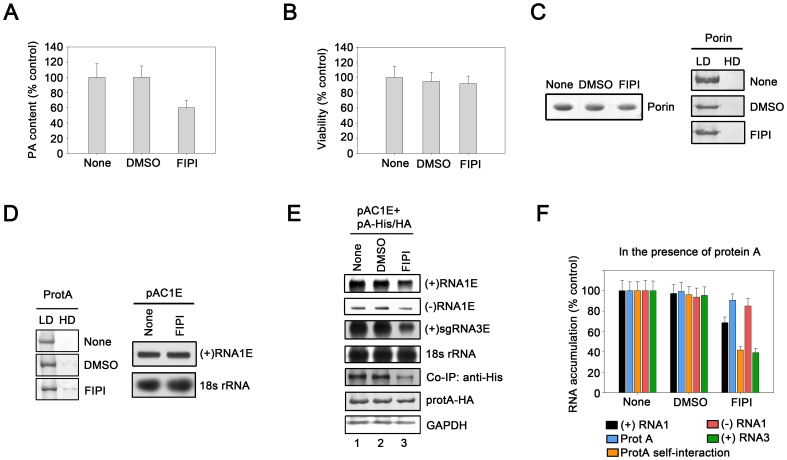
Phospholipids affect the proper functioning of protein A. (A) Measurement of PA content in Pr-E cells or cells treated with 75 nM FIPI or with matching concentration of DMSO (vehicle). (B) Viability of cells treated with FIPI or DMSO. (C) FIPI treatment show less effect on the activity of mitochondrial membrane-binding protein porin to associate with membranes. Left, cells treated with or without FIPI were harvested and then probed via Western blotting with anti-porin antibody. Right, Nycodenz flotation assay were used to examine membrane association of porin in cells treated with FIPI. (D) FIPI treatment show less effect on membrane association of protein A and input plasmid transcription. Right, Nycodenz flotation assay were used to examine membrane association of protein A in cells treated with FIPI. Right, total RNAs was isolated from FIPI treated cells expressing (+)RNA1E templates and then probed via Northern blotting with EGFP and 18s rRNA probes, respectively. (E) RNA accumulation in cells treated with FIPI or DMSO expressing protein A_GAA_-His/HA. Cells were divided into two equal fractions. One of fractions was analyzed via immunoprecipitation with an anti-HA antibody and subjected to Western blotting with anti-His antibody. The other fraction was analyzed via Northern blotting with EGFP and 18s rRNA probes, respectively. Quantification data show the accumulation of (+)RNA1E, (−)RNA1E, and (+)sgRNA3E, and protein A and protein A self-interaction in Pr-E cells expressing protein A–His/HA treated with FIPI or DMSO (F). GAPDH, glyceraldehyde-3-phosphate dehydrogenase. Error bars represent S.D. values from at least three independent experiments and the represent results were shown in (E). The accumulation of RNA and protein is normalized to 18s rRNA and GAPDH, respectively.

We used PA inhibitor FIPI to down-regulate PA in cells [43] because PA is a precursor in the CDP-DAG pathway [7]. Pr-E cells were treated with 75 nM FIPI, which inhibits PA production efficiently and show little negative effect on cells [43]. FIPI treatment yielded a 40% reduction in cellular levels of PA (Fig. 9A). The incomplete blockage of PA production was likely due to the presence of a *de novo* PA synthesis pathway [6], [7]. Moreover, we also assessed cell viability and found that FIPI minimally affected cell viability (∼10% reduction), which was comparable with the effect of the vehicle DMSO (Fig. 9B). The effect of FIPI treatment on mitochondrial associated protein was also assessed via the detection of porin protein, which is an integral membrane protein associated with mitochondria. Our results show that FIPI minimally affected the porin expression (Fig. 9C, left). Moreover, we further determined that FIPI treatment was unable to alter the membrane association of porin via Nycodenz flotation assay (Fig. 9C, right), thereby ruling out the possibility that FIPI treatment can damage the property of mitochondrial membranes to associate with membrane-bound proteins.

Furthermore, we assessed the membrane association of protein A via Nycodenz flotation assay. WhNV protein A was expressed via transfection with plasmid pA. As shown in Fig. 9D, left, FIPI treatment did not alter the activity of protein A to associate with membranes. Moreover, we examined whether inhibiting PA affects the initial transcription from input plasmid. As shown in Fig. 9D, right, the initial transcription from the input plasmid pAC1E was almost the same in cells with or without FIPI treatment.

We examined the effects of MML manipulation on WhNV RNA replication using WhNV *trans*-replication system (Material and Methods). To this end, the cells expressing protein A and RNA1E template were treated with or without FIPI. The accumulations of negative-strand (−)RNA1E, (+)RNA1E and (+)sgRNA3E were determined by Northern blots. The accumulation of (−)RNA1E was only moderately reduced by about 15% in FIPI treated cells compared to that in non-treated cells (Fig. 9E, “(−)RNA1E”, compared lane 3 to lane 2 or 1; Fig. 9F), while the accumulation of (+)sgRNA3E was reduced by about 60% (Fig. 9E, “(+)RNA3E”, compared lane 3 to lane 2 or 1; Fig. 9F). Also, the FIPI treatment resulted in about 30% reduction in the accumulation of (+)RNA1E (Fig. 9E, “(+)RNA1E”, compared lane 3 to lane 2 or 1; Fig. 9F). Moreover, the FIPI treatment showed an apparent biased effect on sgRNA3 production, suggesting that this step may be especially sensitive to inhibiting PA production. Besides, the self-interaction of protein A wt was also inhibited by about 60% via inhibiting PA production (Fig. 9E, “co-IP”, compared lane 3 to lane 2 or 1; Fig. 9F). Although (−)RNA synthesis was less affected, the levels of (+)RNA1 and (+)sgRNA3 were still reduced by the FIPI treatment, indicating that at the similar level of the (−)RNA1 template, the activity of WhNV RdRP to replicate (+)-stranded RNA products was indeed weakened.

## Discussion

RNA replication of (+) RNA viruses requires the association of viral RNA and replicases with intracellular membranes to form vRCs [1]–[3]. To advance the understanding of the relationship between intracellular membranes and viral RNA replicases, we studied the direct effects of membranes, particularly membrane lipids, on the function of the replicase (protein A) from WhNV. We uncover the self-interaction of WhNV protein A and show that this activity of protein A could be stimulated by MMLs. Additional investigations show that MMLs interact with specific fragments of protein A, and this direct lipid-protein interaction may stimulates protein A self-interaction by promoting homotypic and heterotypic interactions of specific fragments. Moreover, the self-interaction of protein A could be selectivity modulated by liposomes generated from specific anionic phospholipids, and specific anionic phospholipids favor different types the homotypic and heterotypic interactions. Furthermore, manipulating phospholipid metabolism via a PA inhibitor weakens protein A self-interaction and RNA replication in cells. Altogether, these findings demonstrate the direct role of membrane lipids in the activity of WhNV protein A.

Two mechanisms may be responsible for the stimulation on protein A self-interaction. One possibility is that MMLs directly mediate protein A activity. The changes in lipid composition may result in protein A’s property changes via altering protein A’s conformation. The other one is that MMLs partition protein A into liposome fraction and thus lead to the increase of protein A’s local density. Binding to common lipid may also contribute to the stimulation on WhNV protein A self-interaction (Fig. 6).

For many (+)RNA viruses, different patterns of protein-protein interactions of replicases are associated with the distinct functions for RNA replication. For example, the 3D polymerase of poliovirus was shown to homooligomerize via two interfaces, which may be related to different function [45], [46]. Similarly, HCV RdRp changes its conformations to direct different function at the early stages of RNA replication [47], [48]. The different homotypic and heterotypic interactions of WhNV protein A provide the direct evidence that the different protein-protein interaction interfaces exist and can be regulated by specific liposomes (Fig. 8). Such different patterns of self-interaction could also be seen from FHV protein A [28]. Although the function of the heterotypic and homotype interactions is not known, it is possible that different interactions may serve to alter the structure, dimerization, or function of protein A, in successive step such as replication complex assembly, RNA replication, and RNA capping. Indeed, when PA production was inhibited in cells, the activity of WhNV protein A to replicate (+)sgRNA3E was preferentially inhibited; however, the synthesis of (−)RNA1E template was minimally affected (Fig. 9F). Because the replication of (−)RNA1E and (+)sgRNA3E are all mediated by protein A, such selective regulation by reducing PA production may be induced by the different homotypic and heterotypic interactions of protein A in response to the changes of membrane lipids.

Membrane lipids are comprised of distinct phospholipids, and the composition of these phospholipids is different for different membranes [7]. It is possible that certain lipids have different effects on (+)RNA replicases. Semliki Forest virus (SFV) localizes to lysosomes and endosomes and the capping activity of SFV NSP1 protein requires association with negative phospholipids PS [42]. Hepatitis C virus (HCV) localizes to membrane lipid rafts and the activity of HCV RdRP requires association with sphingomyelin [49]. According to nodavirus, FHV protein A membrane association [30] and WhNV protein A self-interaction (Figs. 7 and 8) can be mediated by specific anionic phospholipids CL, PA and PG, which are enriched in mitochondrial membranes [7]. In these cases, particular phospholipids enriched in certain intracellular membranes, which are associated with these viruses, show preferential and direct effects on the activities of replicases. However, some universal phospholipids being enriched in many intracellular membranes [7], could also mediate (+)RNA virus replication. For example, PC show less direct impacts on FHV protein A’s membrane association but mediate protein A function in some other ways [30], [33]. These results suggest that the regulations of phospholipids on (+)RNA virus replicase activities could be manifold.

Nonionic detergent (Triton X-100) is preferred for the isolation of membrane proteins, as it assists in the solubilization of proteins from lipids. Then, we used it for the purification of protein A and MMLs. Although we did our best to get rid of the detergent, we can not ensure that all detergents were completely removed. The transformation between liposomes and detergent/lipid mixed micelles is a reversible process that can be induced by the addition or reduction of the concentrations of detergent [50]. Our observation that increasing the concentrations of MMLs enhanced the protein A self-interaction (Fig. 4B) revealed that the concentrations of the remaining detergent is much low or even neglectable. However, it is still possible that the remaining detergents may affect the protein-MML interactions and subsequently weaken the enhanced protein A self-interaction in the presence of MMLs.

Although the *in vitro* data reveals the obvious effects of membrane lipids on WhNV protein A self-interaction (Figs. 4–8), the cellular experiment data shows relatively minor effects (Fig. 9). That may be partly due to that the simplified *in vitro* systems containing only one purified protein and one or a few kinds of lipids, and do not represent the whole behavior of the protein A in replication in cells. In addition, the PA content still remained at 60% level compared to that in cells without FIPI treatment, probably due to the presence of a *de novo* PA synthesis pathway [6], [7]. The production of other anionic phospholipids could be less affected by the FIPI treatment, and may even compensate the lipid loss in mitochondria. Moreover, it is possible that inhibiting the self-interaction of protein A indirectly weakens the viral RNA replication by such as affecting the microenvironment of vRCs or the binding of host factor to vRCs, rather than directly weakens the ability of per unit protein A to synthesize RNA. Furthermore, inhibition of the total cellular PA content in cells may not reflect the real effects of membrane lipids on protein A function. The protein A function can be mediated by multiple factors, such as total cellular lipids content, membrane lipids, and the protein A microenvironment. Our future studies will focus on the effects of other factors, including host proteins or/and other lipids, on WhNV protein A complete activity.

In summary, our findings further reveal the detailed mechanisms by which direct MML-protein interaction regulates the self-interaction of nodaviral replicase protein A. Nodaviral RNA replication is highly parallel with that of other (+)RNA viruses, with regard to the formation of vRCs on host intracellular membranes, the requirement of homo- and/or hetero-oligomerization of viral replicase components (for nodavirus, it is the self-interaction of multiple fragments within the single replicase), and viral RNA replication-associated alterations in the composition of MMLs [28], [29], [33], [51]–[53]. Considering the commonalities that exist between nodaviruses and other (+)RNA viruses in RNA replication, some of the principles revealed in this study may be generally applicable to a range of (+)RNA viruses.

## Supporting Information

Figure S1 **Detection of the purified outer mitochondrial membranes.** The purified outer mitochondrial membranes (OMM) and intact mitochondrial (Mito) was subjected to Western blotting with anti-porin, anti-Tim 23 and anti-Calreticulin, respectively. Tim 23, an inner mitochondrial membrane protein. Calreticulin, an endoplasmic reticulum membrane protein.
(TIF)

## References

[1] Ahlquist P (2006) Parallels among positive-strand RNA viruses, reverse-transcribing viruses and double-stranded RNA viruses. Nat Rev Microbiol 4: 371–382. 16582931 10.1038/nrmicro1389 PMC7097367

[2] Miller S, Krijnse-Locker J (2008) Modification of intracellular membrane structures for virus replication. Nat Rev Microbiol 6: 363–374. 18414501 10.1038/nrmicro1890 PMC7096853

[3] Sasvari Z, Nagy PD (2010) Making of viral replication organelles by remodeling interior membranes. Viruses 2: 2436–2442. 21994625 10.3390/v2112436 PMC3185585

[4] Ahlquist P, Noueiry AO, Lee WM, Kushner DB, Dye BT (2003) Host factors in positive-strand RNA virus genome replication. J Virol 77: 8181–8186. 12857886 10.1128/JVI.77.15.8181-8186.2003 PMC165243

[5] Denison MR (2008) Seeking membranes: positive-strand RNA virus replication complexes. PLoS Biol 6: e270. 18959488 10.1371/journal.pbio.0060270 PMC2573941

[6] Nohturfft A, Zhang SC (2009) Coordination of lipid metabolism in membrane biogenesis. Annu Rev Cell Dev Biol 25: 539–566. 19575637 10.1146/annurev.cellbio.24.110707.175344

[7] van Meer G, Voelker DR, Feigenson GW (2008) Membrane lipids: where they are and how they behave. Nat Rev Mol Cell Biol 9: 112–124. 18216768 10.1038/nrm2330 PMC2642958

[8] Ahlquist P (1992) Bromovirus RNA replication and transcription. Curr Opin Genet Dev 2: 71–76. 1378769 10.1016/s0959-437x(05)80325-9

[9] Palmenberg AC (1990) Proteolytic processing of picornaviral polyprotein. Annu Rev Microbiol 44: 603–623. 2252396 10.1146/annurev.mi.44.100190.003131

[10] Panaviene Z, Baker JM, Nagy PD (2003) The overlapping RNA-binding domains of p33 and p92 replicase proteins are essential for tombusvirus replication. Virology 308: 191–205. 12706102 10.1016/s0042-6822(02)00132-0

[11] Pata JD, Schultz SC, Kirkegaard K (1995) Functional oligomerization of poliovirus RNA-dependent RNA polymerase. Rna 1: 466–477. 7489508 PMC1482417

[12] Reed KE, Rice CM (2000) Overview of hepatitis C virus genome structure, polyprotein processing, and protein properties. Curr Top Microbiol Immunol 242: 55–84. 10592656 10.1007/978-3-642-59605-6_4

[13] Russo M, Burgyan J, Martelli GP (1994) Molecular biology of tombusviridae. Adv Virus Res 44: 381–428. 7817878 10.1016/s0065-3527(08)60334-6

[14] Beckman MT, Kirkegaard K (1998) Site size of cooperative single-stranded RNA binding by poliovirus RNA-dependent RNA polymerase. J Biol Chem 273: 6724–6730. 9506971 10.1074/jbc.273.12.6724

[15] Goregaoker SP, Culver JN (2003) Oligomerization and activity of the helicase domain of the tobacco mosaic virus 126- and 183-kilodalton replicase proteins. J Virol 77: 3549–3556. 12610130 10.1128/JVI.77.6.3549-3556.2003 PMC149526

[16] Qin W, Luo H, Nomura T, Hayashi N, Yamashita T, et al (2002) Oligomeric interaction of hepatitis C virus NS5B is critical for catalytic activity of RNA-dependent RNA polymerase. J Biol Chem 277: 2132–2137. 11673460 10.1074/jbc.M106880200

[17] Wang QM, Hockman MA, Staschke K, Johnson RB, Case KA, et al (2002) Oligomerization and cooperative RNA synthesis activity of hepatitis C virus RNA-dependent RNA polymerase. J Virol 76: 3865–3872. 11907226 10.1128/JVI.76.8.3865-3872.2002 PMC136118

[18] Gallagher TM, Friesen PD, Rueckert RR (1983) Autonomous replication and expression of RNA 1 from black beetle virus. J Virol 46: 481–489. 16789241 10.1128/jvi.46.2.481-489.1983 PMC255150

[19] Schneemann A, Zhong W, Gallagher TM, Rueckert RR (1992) Maturation cleavage required for infectivity of a nodavirus. J Virol 66: 6728–6734. 1404613 10.1128/jvi.66.11.6728-6734.1992 PMC240169

[20] Li H, Li WX, Ding SW (2002) Induction and suppression of RNA silencing by an animal virus. Science 296: 1319–1321. 12016316 10.1126/science.1070948

[21] Ahlquist P (2002) RNA-dependent RNA polymerases, viruses, and RNA silencing. Science 296: 1270–1273. 12016304 10.1126/science.1069132

[22] Venter PA, Schneemann A (2008) Recent insights into the biology and biomedical applications of Flock House virus. Cell Mol Life Sci 65: 2675–2687. 18516498 10.1007/s00018-008-8037-y PMC2536769

[23] Lu R, Yigit E, Li WX, Ding SW (2009) An RIG-I-Like RNA helicase mediates antiviral RNAi downstream of viral siRNA biogenesis in Caenorhabditis elegans. PLoS Pathog 5: e1000286. 19197349 10.1371/journal.ppat.1000286 PMC2629121

[24] Kovalev N, Pogany J, Nagy PD (2012) A Co-Opted DEAD-Box RNA Helicase Enhances Tombusvirus Plus-Strand Synthesis. PLoS Pathog 8: e1002537. 22359508 10.1371/journal.ppat.1002537 PMC3280988

[25] Lu R, Maduro M, Li F, Li HW, Broitman-Maduro G, et al (2005) Animal virus replication and RNAi-mediated antiviral silencing in Caenorhabditis elegans. Nature 436: 1040–1043. 16107851 10.1038/nature03870 PMC1388260

[26] Qiu Y, Cai D, Qi N, Wang Z, Zhou X, et al (2011) Internal initiation is responsible for synthesis of Wuhan nodavirus subgenomic RNA. J Virol 85: 4440–4451. 21325414 10.1128/JVI.02410-10 PMC3126283

[27] Qiu Y, Wang Z, Liu Y, Qi N, Si J, et al (2013) Newly discovered insect RNA viruses in China. Sci China Life Sci 56: 711–714. 23917843 10.1007/s11427-013-4520-2

[28] Dye BT, Miller DJ, Ahlquist P (2005) In vivo self-interaction of nodavirus RNA replicase protein a revealed by fluorescence resonance energy transfer. J Virol 79: 8909–8919. 15994785 10.1128/JVI.79.14.8909-8919.2005 PMC1168736

[29] Miller DJ, Ahlquist P (2002) Flock house virus RNA polymerase is a transmembrane protein with amino-terminal sequences sufficient for mitochondrial localization and membrane insertion. J Virol 76: 9856–9867. 12208963 10.1128/JVI.76.19.9856-9867.2002 PMC136485

[30] Stapleford KA, Rapaport D, Miller DJ (2009) Mitochondrion-enriched anionic phospholipids facilitate flock house virus RNA polymerase membrane association. J Virol 83: 4498–4507. 19244330 10.1128/JVI.00040-09 PMC2668453

[31] Wu SX, Ahlquist P, Kaesberg P (1992) Active complete in vitro replication of nodavirus RNA requires glycerophospholipid. Proc Natl Acad Sci U S A 89: 11136–11140. 1454791 10.1073/pnas.89.23.11136 PMC50504

[32] Wu SX, Kaesberg P (1991) Synthesis of template-sense, single-strand Flockhouse virus RNA in a cell-free replication system. Virology 183: 392–396. 1905080 10.1016/0042-6822(91)90153-3

[33] Castorena KM, Stapleford KA, Miller DJ (2011) Complementary transcriptomic, lipidomic, and targeted functional genetic analyses in cultured Drosophila cells highlight the role of glycerophospholipid metabolism in Flock House virus RNA replication. BMC Genomics 11: 183. 10.1186/1471-2164-11-183 PMC2847973 20236518

[34] Weeks SA, Miller DJ (2008) The heat shock protein 70 cochaperone YDJ1 is required for efficient membrane-specific flock house virus RNA replication complex assembly and function in Saccharomyces cerevisiae. J Virol 82: 2004–2012. 18057252 10.1128/JVI.02017-07 PMC2258711

[35] Qi N, Zhang L, Qiu Y, Wang Z, Si J, et al (2012) Targeting of dicer-2 and RNA by a viral RNA silencing suppressor in Drosophila cells. J Virol 86: 5763–5773. 22438534 10.1128/JVI.07229-11 PMC3347268

[36] Qi N, Cai D, Qiu Y, Xie J, Wang Z, et al (2011) RNA binding by a novel helical fold of b2 protein from wuhan nodavirus mediates the suppression of RNA interference and promotes b2 dimerization. J Virol 85: 9543–9554. 21734038 10.1128/JVI.00785-11 PMC3165748

[37] Wang Z, Qiu Y, Liu Y, Qi N, Si J, et al.. (2013) Characterization of a Nodavirus Replicase Revealed a De Novo Initiation Mechanism of RNA Synthesis and Terminal Nucleotidyl Transferase Activity. J Biol Chem. 10.1074/jbc.M113.492728 PMC3829395 24019510

[38] Qiu Y, Wang Z, Liu Y, Qi N, Miao M, et al (2013) Membrane association of Wuhan nodavirus protein A is required for its ability to accumulate genomic RNA1 template. Virology 439: 140–151. 23490047 10.1016/j.virol.2013.02.010

[39] Han X, Ying X, Huang HV, Zhou S, Huang Q (2012) Expression and purification of enterovirus type 71 polyprotein P1 using Pichia pastoris system. Virol Sin 27: 254–258. 22899434 10.1007/s12250-012-3256-7 PMC8218036

[40] Graham JM (1993) Isolation of mitochondria, mitochondrial membranes, lysosomes, peroxisomes, and Golgi membranes from rat liver. Methods Mol Biol 19: 29–40. 8220704 10.1385/0-89603-236-1:29

[41] Graham JM (2001) Isolation of mitochondria from tissues and cells by differential centrifugation. Curr Protoc Cell Biol Chapter 3: Unit 3 3. 10.1002/0471143030.cb0303s04 18228355

[42] Ahola T, Lampio A, Auvinen P, Kaariainen L (1999) Semliki Forest virus mRNA capping enzyme requires association with anionic membrane phospholipids for activity. Embo J 18: 3164–3172. 10357827 10.1093/emboj/18.11.3164 PMC1171397

[43] Su W, Yeku O, Olepu S, Genna A, Park JS, et al (2009) 5-Fluoro-2-indolyl des-chlorohalopemide (FIPI), a phospholipase D pharmacological inhibitor that alters cell spreading and inhibits chemotaxis. Mol Pharmacol 75: 437–446. 19064628 10.1124/mol.108.053298 PMC2684902

[44] Hojjati MR, Jiang XC (2006) Rapid, specific, and sensitive measurements of plasma sphingomyelin and phosphatidylcholine. J Lipid Res 47: 673–676. 16371647 10.1194/jlr.D500040-JLR200

[45] Hansen JL, Long AM, Schultz SC (1997) Structure of the RNA-dependent RNA polymerase of poliovirus. Structure 5: 1109–1122. 9309225 10.1016/s0969-2126(97)00261-x

[46] Lyle JM, Bullitt E, Bienz K, Kirkegaard K (2002) Visualization and functional analysis of RNA-dependent RNA polymerase lattices. Science 296: 2218–2222. 12077417 10.1126/science.1070585

[47] Biswal BK, Cherney MM, Wang M, Chan L, Yannopoulos CG, et al (2005) Crystal structures of the RNA-dependent RNA polymerase genotype 2a of hepatitis C virus reveal two conformations and suggest mechanisms of inhibition by non-nucleoside inhibitors. J Biol Chem 280: 18202–18210. 15746101 10.1074/jbc.M413410200

[48] Chinnaswamy S, Yarbrough I, Palaninathan S, Kumar CT, Vijayaraghavan V, et al (2008) A locking mechanism regulates RNA synthesis and host protein interaction by the hepatitis C virus polymerase. J Biol Chem 283: 20535–20546. 18442978 10.1074/jbc.M801490200 PMC2459299

[49] Weng L, Hirata Y, Arai M, Kohara M, Wakita T, et al (2010) Sphingomyelin activates hepatitis C virus RNA polymerase in a genotype-specific manner. J Virol 84: 11761–11770. 20844041 10.1128/JVI.00638-10 PMC2977884

[50] Seddon AM, Curnow P, Booth PJ (2004) Membrane proteins, lipids and detergents: not just a soap opera. Biochim Biophys Acta 1666: 105–117. 15519311 10.1016/j.bbamem.2004.04.011

[51] Kopek BG, Settles EW, Friesen PD, Ahlquist P (2010) Nodavirus-induced membrane rearrangement in replication complex assembly requires replicase protein a, RNA templates, and polymerase activity. J Virol 84: 12492–12503. 20943974 10.1128/JVI.01495-10 PMC3004334

[52] Van Wynsberghe PM, Chen HR, Ahlquist P (2007) Nodavirus RNA replication protein a induces membrane association of genomic RNA. J Virol 81: 4633–4644. 17301137 10.1128/JVI.02267-06 PMC1900146

[53] Miller DJ, Schwartz MD, Ahlquist P (2001) Flock house virus RNA replicates on outer mitochondrial membranes in Drosophila cells. J Virol 75: 11664–11676. 11689648 10.1128/JVI.75.23.11664-11676.2001 PMC114753

