# Supplementary figures and images for: The Self-Interaction of a Nodavirus Replicase Is Enhanced by Mitochondrial Membrane Lipids
Source: PLoS One. 2014 Feb 25;9(2):e89628. doi: 10.1371/journal.pone.0089628 (PMC3934934; doi:10.1371/journal.pone.0089628)

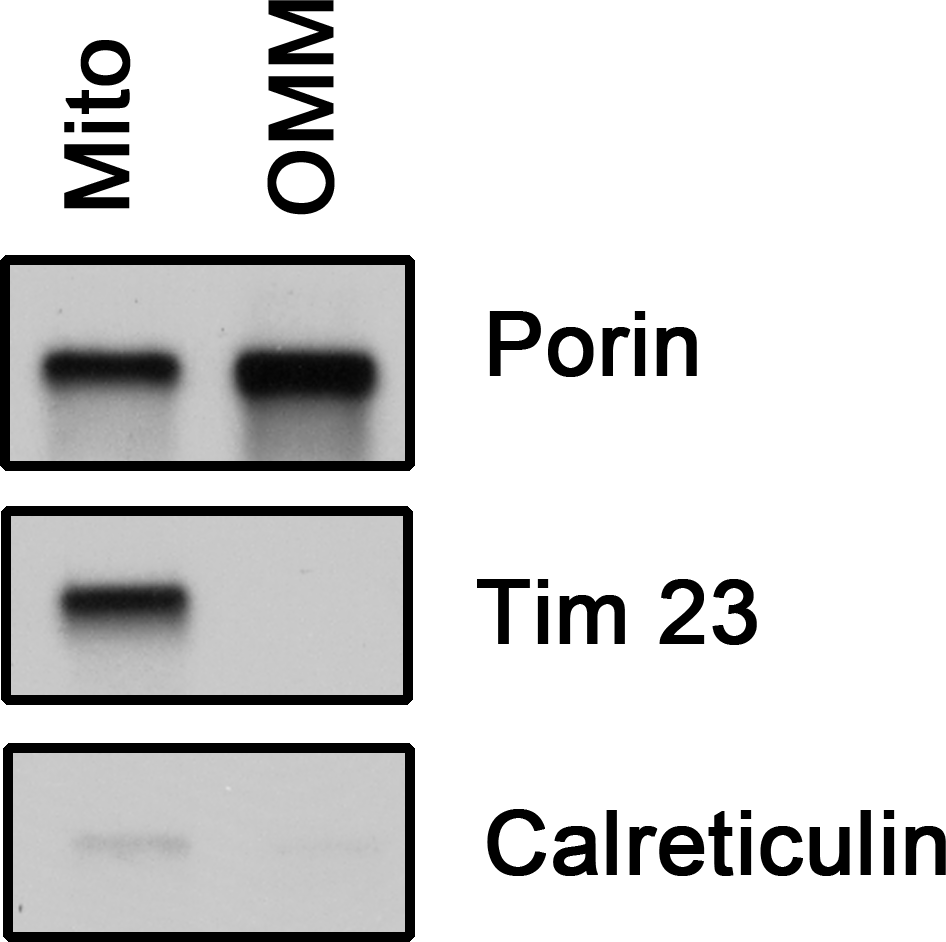

Supplement: Figure S1 — Detection of the purified outer mitochondrial membranes. The purified outer mitochondrial membranes (OMM) and intact mitochondrial (Mito) was subjected to Western blotting with anti-porin, anti-Tim 23 and anti-Calreticulin, respectively. Tim 23, an inner mitochondrial membrane protein. Calreticulin, an endoplasmic reticulum membrane protein. (TIF) [file pone.0089628.s001.tif]
